# Gastric aspiration in sudden unexpected infant death of Prader–Willi syndrome: immunohistochemical detection of feeding components

**DOI:** 10.1007/s00414-022-02883-1

**Published:** 2022-08-26

**Authors:** Motoki Osawa, Haruka Ikeda, Atsushi Ueda, Haruaki Naito, Ryoko Nagao, Yu Kakimoto

**Affiliations:** Department of Forensic Medicine, Tokai University School of Medicine, Isehara, Kanagawa Japan

**Keywords:** β-lactoglobulin, Gastroesophageal reflux, Immunohistochemistry, Milk formula, Tube feeding

## Abstract

Prader–Willi syndrome (PWS) in infants is characterized by hypotonia and poor sucking with feeding difficulties. Two autopsy cases of sudden unexpected death during sleep after tube feeding are described herein. For one, gastric aspiration caused by the possible milk regurgitation was suspected. Immunohistochemical examination of lung sections was performed using three antibodies to human α-lactalbumin, human gross cystic disease fluid protein 15, and cow whey β-lactoglobulin. Five cases of sudden unexpected infant death occurring earlier than at 6 months old were selected as controls. Marked immune-staining for infant formula in one PWS subject was evident within terminal bronchioles and alveoli with granular and amorphous features. However, no positive staining was apparent in the other subject, who exhibited contrasting features in milk distribution. Among control cases, one showed mild staining in the bronchiole, but the others did not. The antibody to β-lactoglobulin reacted specifically with formula, with no nonspecific background. Gastric contents in the airway can be a difficult issue because of the consequent terminal gasping. However, because of an episode of antemortem symptoms of potential regurgitation, and from findings at autopsy such as petechiae, we inferred that fatal regurgitation occurred in this PWS infant after tube feeding. Several clinical reports have described milk aspiration, but this pathological report is the first related to aspiration in PWS during tube feeding.

## Introduction

An infant with Prader–Willi syndrome (PWS) is characterized by neonatal muscular hypotonia and poor sucking with feeding difficulties that require nasogastric tube placement for nutrition: a so-called floppy infant. After failure to thrive in early infancy, affected individuals develop an insatiable appetite, hyperphagia, and obesity during childhood [1]. Growth hormone treatment has improved patients’ body composition and prognosis, but many PWS patients die suddenly of respiratory and cardiovascular causes at all ages [2–4]. This genetic disorder, PWS, is caused by the lack of expression of paternal genes on the long arm of chromosome 15, either because of paternal chromosomal deletion or maternal disomy [5].

Milk and oral secretions are the most frequent foreign materials causing pulmonary aspiration in children [6]. Particularly, impairment of weak or uncoordinated swallowing increases risk of respiratory complications in PWS patients [7, 8]. Symptoms of aspiration are usually subtle. The affected patients might display increased noisy breathing during or after feeding [9]. Sudden unexpected death occurs during infancy. For such cases, suspected milk aspiration has been recorded in several reports [10–13].

However, in a PWS infant case with tube feeding, aspiration from regurgitation of gastric contents is expected to be more plausible than direct aspiration from impaired oral swallowing [14]. The inhalation of vomitus including acid, gastric particles, and the mixture of stomach contents engenders acute lung injury, clinically designated as acute respiratory distress syndrome, in addition to obstruction of the narrow airway [15].

Postmortem evaluation of gastric aspiration is not easy for a couple of reasons. Gastric contents in the airway can be a consequence of terminal gasping during physiological gastroesophageal reflux [16]. Alex et al. showed that aspiration of agonal or postmortem artifact was observed only to a mild degree among sudden infant death syndrome (SIDS) cases, suggesting that the features have limited relevance to the mechanism of suffocation [17]. Another reason is that such gastric aspiration might be enhanced during artificial cardiopulmonary resuscitation (CPR) attempts at the scene and hospital [18]. However, almost every infant is transferred to an emergency hospital, even in a situation of cardiac arrest, where the infant subsequently receives CPR [17]. Forensic pathologists often encounter the dilemma of antemortem fatal aspiration or a secondary terminal event.

As described herein, we examined two cases of sudden unexpected infant death (SUID) of PWS. The infants of these cases, who were found unconscious during sleep after tube feeding, exhibited quite contrasting features in postmortem examinations. To investigate the possibility of gastric aspiration, we attempted to detect milk components using immunohistochemical analysis. After presenting the results of our investigation, we can discuss their meanings.

## Case presentation

Two cases of SUID occurring in PWS infants are described below. Major findings of postmortem examinations are presented in Table 1.

**Table 1:** Detection of milk components in the lung sections of PWS and the control subjects

No	Age	CPR (h)^a^	Position at scene	Diagnosis	Milk under microscopy^b^		Milk in gross observation		Petechiae^d^		
					Bronchiole	Alveoli	Stomach	Esophagus	Thymus	Heart	Lung
PWS#1	5 m	0.5	Supine	Aspiration	+ +	+ +	+ +	+	+ +	+	+ +
PWS#2	3 m	5	Supine	SIDS	-	-	-	-	-	-	-
C#1	2 m	1	Supine	Unknown	+	-	± ^c^	-	+ +	+	+
C#2	1 m	No	Supine	SIDS	-	-	+	-	-	+	+
C#3	4 m	0.5	Prone	SIDS	-	-	±	-	+	-	+
C#4	4 m	1.5	Supine	SIDS	-	-	-	-	-	+	+ +
C#5	2 m	0.5	Supine	Unknown	-	-	-	-	+	+	+ +

^a^CPR means the time (h) of arrival to declaration of death at hospital. The exact time for CPR attempt was not clear at all

^b^The degrees of milk aspiration were classified as − , absent, + , mild, and +  + , highly based on staining in alveoli, according to an earlier report by Krous et al. [16]

^c^A tiny amount of less than a small spoon

^d^Petechiae bleeding in the serosal membrane, and the milk volume in the stomach and esophagus were classified into three grades from absent ( −) to highly (+ +), based on gross observation

Case PWS#1: A 5-month-old male infant had been born by caesarean section at the full term of gestation, with birth weight of 2,144 g. He had respiratory distress, hypotonia, and feeding failure after birth, for which he was admitted to the neonatal intensive care unit (NICU) for 2 months. PWS was diagnosed based on genetic testing as deletion in the *SNRPN*-containing region of the father’s chromosome 15. His mother fed him formula through a nasogastric tube at home. Two days before his death, she noticed hard breathing and noisy throat in the baby. She took him to a pediatrician. Two days later, in the early morning, he was found unresponsive in the supine position at home. Then, he was taken to an emergency hospital. He was in cardiopulmonary arrest on arrival. He died 30 min after arrival at the hospital while undergoing CPR.

Case PWS#2: A 3-month-old female infant, who had been delivered vaginally at 34 weeks, with birth weight of 2690 g. PWS was suspected from a low Apgar score, feeding failure, and hypotonia and was subsequently diagnosed from genetic testing as having paternal deletion. After hospitalization in the NICU for 6 weeks, she had a seizure at home. However, an electroencephalogram yielded no abnormal finding. One day around noon, 3 h after feeding her baby through a gastric tube, the mother found the baby to be unresponsive during sleep. She rushed her baby to the emergency room. The baby was in cardiopulmonary arrest on arrival, but CPR treatment restored her heartbeat temporarily. Finally, she died 5 h after arriving at the hospital.

As control subjects, we selected five cases of SUID involving infants younger than 6 months old who were compatible to the two subjects. The PWS and control subjects described herein were autopsied in our department within 2 days after death.

## Findings at autopsy and in histopathology

The series of postmortem examinations was performed extensively as reported earlier [19]. Briefly, full autopsy was conducted with tissue examination of formalin-fixed organs using hematoxylin–eosin (H&E) staining under microscopy, including special staining, such as Gram staining. Biochemical laboratory tests were applied for a couple of markers that are stable at postmortem. However, only a few test items such as C reactive protein (CRP) were examined because of the limited amount of serum samples obtained from autopsy. Viral titer including specific antibody was measured in the serum for adenovirus types 3 and 7, influenza A and B viruses, respiratory syncytial virus, coxsackievirus B1–B6, and cytomegalovirus IgG and IgM [20]. Blood culture was also performed for screening for pathogenic bacteria. Furthermore, we routinely check genome sequence for major genes regarded as causes of cardiovascular diseases such as QT prolongation, Brugada syndrome, arrhythmogenic right ventricular cardiomyopathy, and hypertrophic cardiomyopathy in the Ion PGM system (Thermo Fisher Scientific Inc.) [21].

**Fig. 1:**
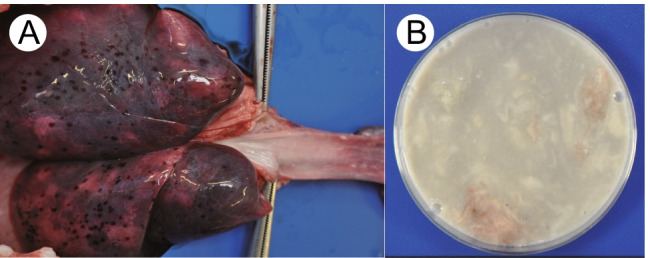
Lumen of his lower esophagus and the back side of his lungs (**A**), and the removed gastric contents (**B**) in gross observation of PWS#1 case. Both contained the whitish aggregate of milk. In **A**, many large spotty bleedings are also evident on the pleura

Case PWS#1: Gross findings at autopsy included many petechiae in the thymus, epicardium, and pleura. Coagulated milk was found in the lower part of the esophagus, as well as in the stomach (Fig. 1). Small whitish aggregates were present in the lung bronchi. Tonsils and cervical lymph nodes were swollen. Histological examination under a microscope showed increased lymphatic follicles in the tonsils and hemophagocytic features in the cervical lymph nodes. Neutrophil infiltration was observed in the spleen. The level of CRP was 0.2 mg/dl. All examined viruses were negative. Genetic testing detected no predictable pathogenic mutation for cardiovascular diseases.

Case PWS#2: At autopsy, no marked change was found except for congestion in the organs. No apparent petechiae were observed in the thymus, epicardium, or pleura. The stomach was empty. No milk aggregate was present in the esophagus. The lymph nodes were not swollen. Inflammatory cell proliferation in the spleen was not observed, CRP was 0.3 mg/dl. Titers were below the reference level for all viruses. No pathogenetic mutation was detected in candidate genes of cardiovascular diseases.

## Immunohistochemical analysis

In an earlier study, we detected breast milk and infant formula components using specific antibodies [22]. Immunohistochemical analysis was applied to lung tissues. At least four specimens were obtained, including one each from the upper left, lower left, upper right, and lower right lobe. Deparaffinized sections of 3-µm thickness were prepared. Immunological reactions were tested using the following antibodies to human α-lactalbumin (Dako, Agilent Corp., USA), human gross cystic disease fluid protein 15 (GCDFP15) (Dako), and cow whey protein β-lactoglobulin (Morinaga Milk Industry Co. Ltd., Yokohama, Japan). Iwadate et al. [23] recommended the antibody for human α-lactalbumin for immunohistochemical analysis to detect milk. Immunostaining was conducted in the EnVision Flex system (Dako) using diaminobenzidine as the substrate for peroxidase, as described earlier [24].

Immunohistochemical analysis was applied to sections of the lungs. The staining features were compatible among the three antibodies used. Antibodies to α-lactalbumin and GCDFP15 antibodies reacted with both breast milk and infant formula, accompanied by cross-reactive positive staining like mucus cells, and mild background. By contrast, anti-β-lactoglobulin antibody reacted only with formula, but the reaction was specific with no background in the specimens. For milk, particularly formula, β-lactoglobulin yielded the best result.

**Fig. 2:**
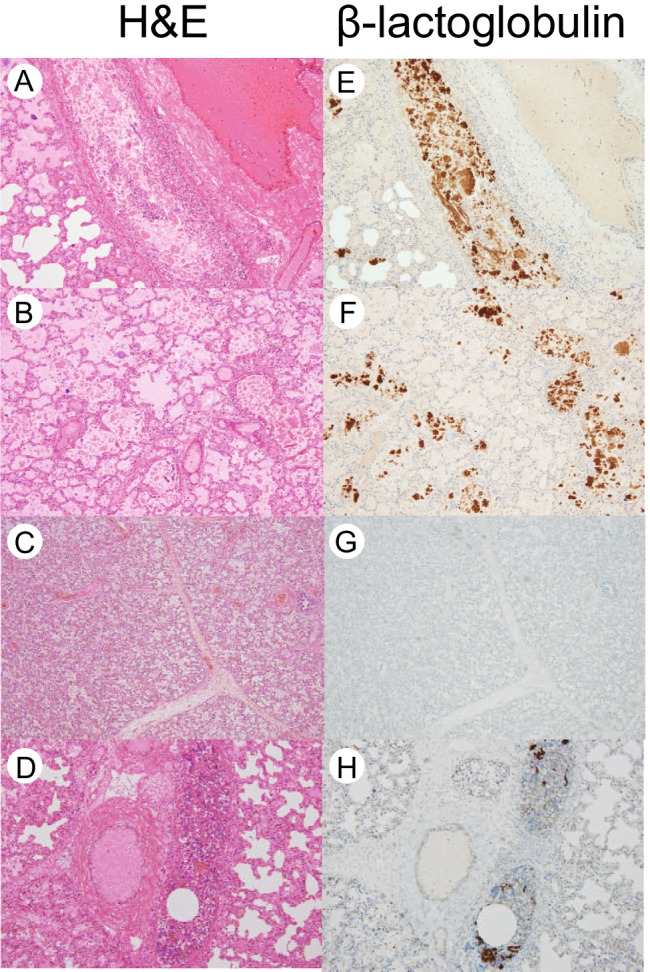
H&E staining (**A**–**D**) and immunohistochemistry using anti-β-lactoglobulin antibody (**E**–**H**) to the lung sections for the PWS and control subjects: **A** and **E**, bronchiole in PWS#1 (× 100); **B** and **F**, alveoli in PWS#1 (× 100); **C** and **G**, PWS#2 (× 40); and **D** and **H**, control #1 (× 100). The left H&E staining and the other right immunostaining are serial sections

As presented in Fig. 2, marked immunostaining for the feeding component was evident within terminal bronchiole and alveolar spaces with granular and amorphous patterns for case PWS#1. Foreign materials were identified with eosinophilic granules in serial sections by H&E staining. By contrast, no apparent positive staining was observed in the lung sections associated with case PWS#2.

For the five control cases, one showed positive, with mild staining only in the bronchiole of lungs; the others did not. In three cases including the positive one, small volumes of milk aggregates were present in the stomach, but not in their esophagus. The degrees of major findings are presented in Table 1.

## Discussion

Because gastric aspiration was suspected for one of the two SUID cases of PWS, the presence of milk in the airway was examined using immunohistochemical analysis with antibodies specific to the components. The infant in case PWS#1 was found to have a large amount of formula in the bronchioles and alveoli of the lungs, but none was found for case PWS#2.

For case PWS#1, formula components were detected widely in bronchioles and in alveoli. The distribution was much deeper than that of the positive case C#1 in the control group. Therefore, the features in PWS case were unlikely to be an artifact caused by agonal gastroesophageal reflux. The mother might have noticed regurgitation sounds in the infant’s throat with a visit to hospital. The infant should suffer from abnormal swallowing or regurgitation in tube feeding. There was also extreme petechial bleeding. Based on the observations described above, it can be inferred that muscular hypotonia was associated with the lethal event. Mohammed et al. reported that nasogastric intubation led to aspiration pneumonia as a result of gastric dilatation [25]. We inferred that regurgitation of gastric contents occurred in this PWS#1 infant under tube feeding.

In general, gastric contents are inhaled into the lungs of 12–40% of infants whose deaths are attributed to SIDS or SUID [14, 26]. This spontaneous aspiration caused by gastroesophageal reflux, including steps of relaxation of esophageal sphincters, suppression of upper airway protective reflexes, and terminal inspiratory efforts, occurs even among non-resuscitated corpses as a terminal event [27]. Terminal gasping and autoresuscitation are thought to generate aspiration from the gastroesophageal route [14, 28, 29]. It is noteworthy that the presence of gastric contents in the airway does not invariably imply the occurrence of antemortem fatal aspiration [26].

The presence of milk in the lungs was also observed in one among the five control SUID cases. In this C#1 case, because a tiny volume of milk was present in the stomach, secondary gastric aspiration can be expected to have occurred in the agonal or postmortem phase. In accordance with the evidence obtained in a previous series of SIDS autopsy cases [17, 26], the degree of aspiration was limited to a mild degree. The sleeping position at the scene reportedly was supine for the two cases of milk aspiration: PWS#1 and C#1. However, no apparent difference of the risk of gastric aspiration into the upper airway and lungs has been confirmed by placing infants in either a supine or prone sleeping position [30].

Formula could not be confirmed in bronchioles or pulmonary alveoli of the PWS#2 infant. Resuscitation was performed for 5 h. Judging whether milk aspiration occurred at the time of death is difficult. Krous et al. [15] pointed out that the aspiration observation is not apparent in infants that had achieved resuscitation for over 48 h. However, it is unlikely that milk would become undetectable within about 5 h. Therefore, the case is judged as sudden death that had occurred by a mechanism other than aspiration. Central apnea has been known to occur in PWS [31, 32], but confirming central apnea is difficult based solely on postmortem examinations. Since no other cause was identifiable, SIDS is suspected to have occurred in this PWS#2 case.

Identifying ingested milk coagulates in the airway solely based on morphology in H&E-stained sections was not easy. For immunohistochemical analysis of milk, the use of α-lactoalbumin antibody has been recommended [22]. Maiese et al. [33] verified milk aspiration from results of immunohistochemistry. However, this anti-α-lactoalbumin antibody was unavailable. The manufacturer discontinued the product. This study used anti-GCDFP15. This antibody also reacted with both breast milk and infant formula, producing comparable staining patterns. In clinical practice, the breast cancer marker GCDFP15 antibody is used routinely with reaction conditions that have been already set by laboratories. These antibodies are useful for milk screening, but an important shortcoming related to their use is that they also react with, for example, goblet cells that secrete mucin.

Our earlier study demonstrated that β-lactoglobulin antibody is effective for detecting infant formula [22]. The reaction is specific. Moreover, it has the least non-specific background in these immunohistochemical examinations. However, it is a shortcoming that this antibody reacts only with formula, but not with human breast milk [34]. Results of this study also support the usefulness of this antibody for the detection of infant formula.

In conclusion, two contrasting cases of PWS have been presented herein. In one case, gastric aspiration was confirmed from immunohistochemical examination using antibodies to milk and formula components, which were effective to prove their presence in the airway. Results indicate that gastric milk can be aspirated into the airway during and after tube feeding in PWS infants.
